# Combination and sequential treatments in the management of osteoporosis

**DOI:** 10.1186/ar3718

**Published:** 2012-03-08

**Authors:** Socrates Papapoulos

**Affiliations:** 1 Department of Endocrinology & Metabolic Diseases, Leiden University Medical Center, Leiden, The Netherlands

## 

Therapies of chronic diseases should be efficacious, convenient for the patient and devoid of side effects. In daily practice, the risk of serious outcomes and the preference of patients as well as the cost of the interventions should also be considered. The pathophysiological basis of osteoporosis provides the rationale for the use of interventions that either reduce bone resorption and turnover or stimulate bone formation. Several antiresorptive treatments are used in the treatment of osteoporosis but PTH is the only anabolic therapy currently available. Evidence for efficacy and safety from controlled studies has been obtained for up to 10 years for antiresorptives and up to 2 years for PTH. while short-term head-to-head studies with surrogate endpoints have also been performed. Such studies illustrate the different mechanism of action of the two types of interventions but do not allow any conclusions about any potential differences in antifracture efficacy. These considerations are reflected in recommendations of several regulatory authorities. It is also frequently assumed that antiresorptives should be given mainly to patients with high bone turnover while anabolics should be reserved for patients with low bone turnover. However, analyses of the results of trials with bisphosphonates and PTH 1-34 indicated that the antifracture efficacy of these agents is independent of prevalent rates of bone turnover. Further analysis of the pharmacodynamic responses to these treatments, reveal distinct patterns with attainment or not of steady-states that provide the basis for the design of regimens with the use of both types of therapies, in some patients at least. Most of the studies have been performed with bisphosphonates and PTH. Combination therapies, a common approach in the treatment of other chronic disease, do not confer any particular advantage compared to monotherapies, although the response may depend on the frequency of the administration of the bisphosphonate. In contrast, sequential therapies are very important for clinical practice and depend on the severity of the disease and the mechanism of action of the specific treatment. Such therapeutic approaches need to be explored further and their efficacy in reducing fracture risk, their safety as well as their cost-effectiveness need to be evaluated.

